# Phase 1 study of the protein deubiquitinase inhibitor VLX1570 in patients with relapsed and/or refractory multiple myeloma

**DOI:** 10.1007/s10637-020-00915-4

**Published:** 2020-03-03

**Authors:** Eric K. Rowinsky, Agne Paner, Jesus G. Berdeja, Claudia Paba-Prada, Parameswaran Venugopal, Kimmo Porkka, Joachim Gullbo, Stig Linder, Angelica Loskog, Paul G. Richardson, Ola Landgren

**Affiliations:** 1 Vivolux AB, Uppsala Science Park, c/o NEXTTOBE AB, Dag Hammarskjölds väg 40c, SE-751 83 Uppsala, Sweden; 2 Division of Hematology, Department of Internal Medicine, Rush University Cancer Center, Chicago, IL USA; 3 Division of Hematology and Oncology, Sarah Cannon Research Institute, Nashville, TN USA; 4 Jerome Lipper Multiple Myeloma Center, Department of Medical Oncology, Dana-Farber Cancer Institute, Boston, MA USA; 5 Hematology Research Unit Helsinki, University of Helsinki, Helsinki, Finland; 6 Department of Hematology, Helsinki University Hospital Comprehensive Cancer Center, Helsinki, Finland; 7 Department of Oncology-Pathology, Karolinska Institute, Stockholm, Sweden; 8 Department of Medical and Health Sciences, University of Linköping, Linköping, Sweden; 9 Department of Immunology, Genetics and Pathology, Uppsala University, Uppsala, Sweden; 10 Myeloma Service, Department of Medicine, Memorial Sloan Kettering Cancer Center, New York, NY USA

**Keywords:** VLX1570, Multiple myeloma, Protein deubiquitinase inhibitor, 19S proteasome, Pulmonary toxicity

## Abstract

This phase 1 study sought to characterize the safety, tolerability, and pharmacokinetic behavior of VLX1570, a small molecule inhibitor of the deubiquitinases (DUBs) that remove sterically bulky ubiquitin chains from proteins during processing in the19S regulatory subunit of the proteasome, in patients with relapsed and refractory multiple myeloma (MM). Fourteen patients were treated with escalating doses of VLX1570 ranging from 0.05 to 1.2 mg/kg as a brief intravenous (IV) infusion on Days 1, 2, 8, 9, 15, and 16 of a 28-day cycle. Due to its poor aqueous solubility, VLX1570 was formulated in polyethylene glycol, polyoxyethylated castor oil, and polysorbate 80 and administered as a brief intravenous (IV) infusion via a central venous catheter. Anti-myeloma effects were noted at doses at or above 0.6 mg/kg, however, two patients treated at the 1.2 mg/kg dose level experienced severe, abrupt, and progressive respiratory insufficiency, which was associated with diffuse pulmonary infiltrates on imaging studies, similar to those rarely noted with bortezomib and other inhibitors of the 20S proteasome, culminating in death. Although the contribution of VLX1570’s formulation to the pulmonary toxicity could not be ruled out, the severity and precipitous nature of the toxicity and the steep relationship between dose and toxicity, the study was discontinued. Despite the severe pulmonary toxicity noted with VLX1570, efforts directed at identifying DUB inhibitors with greater therapeutic indices appear warranted based on the unique mechanism of action, robustness of preclinical antitumor activity, and activity of the DUB inhibitors in MM resistant to PIs targeting the 20S proteasome subunit.

## Introduction

**Fig. 1:**
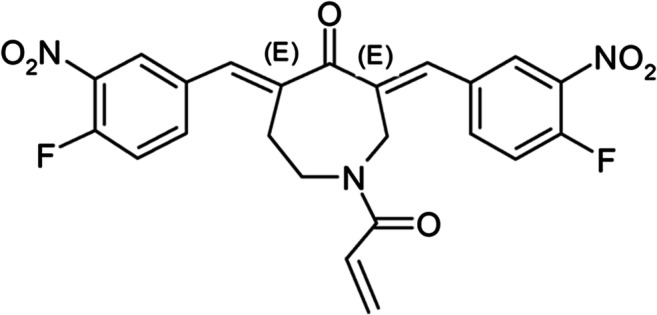
Chemical Structure of VLX1570

Targeting proteasome function is a principal therapeutic pillar in the treatment of multiple myeloma (MM) and other plasma cell dyscrasias [1–3]. In the current era of clinical medicine, most patients diagnosed with MM are typically treated with at least one proteasome inhibitor (PI), but drug resistance invariably develops [2, 3]. Currently available proteasome inhibitors, including the prototypical PI bortezomib and next generation PIs, carfilzomib, ixazomib, and others, target the 20S proteasome subunit, in which proteins encounter the proteolytic active site of the proteasome and undergo degradation [1–3]. The central core of the 20S subunit is narrow and gated by the N-terminal tails of the α ring subunits, thereby requiring protein substrates to be at least partially unfolded before entering the core [1, 3] The dynamic process of protein unfolding before entering the core is facilitated by deubiquitinases (DUBs), which remove sterically bulky ubiquitin chains in the 19S regulatory subunit of the proteasome [4–11]. The small molecule VLX1570 (Fig. 1) and an analog b-AP15 specifically block the activity of DUBs USP14 and UCHL5 in the 19S regulatory subunit, which results in the rapid accumulation of high molecular weight ubiquitin conjugates, proteasome shutdown, and robust anti-tumor activity in well-established orthotopic and xenograft models of MM, lymphoma, Ewing’s sarcoma, and other malignancies [4–11]. In addition to demonstrating robust cytotoxic activity and cellular responses like bortezomib in preclinical models of MM, VLX1570 possessed prominent activity in bortezomib-resistant MM [9–11]. In rats, the maximum tolerated dose (MTD) of VLX1570, which required formulation in polyethylene glycol, polyoxyethylated castor oil, and polysorbate 80 in due to its poor aqueous solubility, was 3.3 mg/kg (20 mg/kg) as a 10-min intravenous (IV) infusion on two consecutive days weekly for 4 weeks.^12^ The highest dose investigated, 6.6 mg/kg caused transient dyspnea, minimal to slight hyperplasia of the alveolar epithelium, and mortality in 3 of 15 rats [12]. There were no findings indicative of respiratory distress as a cause of mortality at that time. In cynomolgus monkeys, the no adverse effect level (NOAEL) was the highest feasible dose of 2.2 mg/kg, with higher doses (3.3 and 6 mg/kg subcutaneously and IV) associated with severe injection site reactions due to the formulation. Other notable effects included transient cytopenia and mild hemolysis attributed to polysorbate 80. In both species, VLX1570 was rapidly distributed and cleared from plasma with mean elimination half-life values ranging from 5 to 8 min, which was predicted by the extensive and rapid drug metabolism observed in ex vivo hepatocyte and animal studies. The unique mechanism of action, activity in bortezomib-resistant MM, and tolerability of VLX1570 provided the rationale to initiate clinical development in relapsed and refractory MM.

Here, we report our experience with single-agent VLX1570 administered IV over 10–30 min on Days 1, 2, 8, 9, 15, and 16 of a 28-day cycle in a multicenter phase 1 study (NCT02372240). The primary objective was to determine the MTD of VLX1570 on this administration schedule and recommend a dose for subsequent phase 2 studies in patients with relapsed and refractory MM and other relevant malignancies. Secondary objectives were to characterize the adverse events and safety profile of VLX1570, its pharmacokinetic (PK) behavior, and evaluate preliminary evidence of anti-cancer activity.

## Patients and methods

This study, which was performed at five institutions in the United States of America and Finland, was comprised of two stages. Stage 1 was a dose-escalation stage, in which the safety and tolerability of VLX1570 was assessed to recommend a dose for subsequent Phase 2 studies disease directed studies, whereas Stage 2 was planned as a Phase 2 study, in which as many as 26 patients were to receive treatment at the recommended phase 2 dose established in Stage 1. Toxicity was graded according to the National Cancer Institute Common Terminology Criteria for Adverse Events, version 4.0. Response was assessed by International Myeloma Working Group criteria after each cycle [13].

Principal eligibility criteria included patients with MM who had been refractory to or relapsed after treatment with at least one immunomodulatory drug (IMiD) and one PI. Patients were required to have: an Eastern Cooperative Oncology Group performance status of 0–2, age ≥ 18 years; measurable disease, as defined by at least one of the following: serum monoclonal protein ≥0.5 g/dL; urine monoclonal protein >200 mg/24 h; and/or serum immunoglobulin free light chain >10 mg/dL and an abnormal kappa/lambda ratio (reference range, 0.26–1.65). All patients had to have adequate bone marrow reserves (defined as absolute neutrophil count ≥1000/mm^3^, platelets ≥75,000/mm^3^, hemoglobin ≥8 g/dL), hepatic function (bilirubin ≤1.5 times the upper limit of normal [ULN], alanine aminotransferase and aspartate aminotransferase values ≤2.5 times the ULN) and renal function (estimated glomerular filtration rate ≥ 30 mL/min). Female patients must have been of non-childbearing potential or have had a negative serum pregnancy test and both male and female patients must have been willing to use effective contraception throughout the study and for 6 months following last study treatment. All patients must have been willing to provide informed written consent according to institutional guidelines.

VX1570 was formulated in polyethylene glycol, polyoxyethylated castor oil, and polysorbate 8. 0Based on observations in the rat toxicity study and the inability to determine an MTD in monkeys due to VLX570’s formulation, the agent was administered by a central venous catheter and respiratory and cardiovascular functions were monitored in the peri-treatment period. The premedication regimen, largely instituted based on the nature of the formulation consisted of: dexamethasone 20 mg orally/IV, diphenhydramine 50 mg oral/IV or an equivalent H_1_-histamine antagonist, and an H_2_-antagonist (cimetidine 300 mg oral/IV, ranitidine 50 mg oral/IV, or famotidine 20 mg IV).

In accordance with Food and Drug Administration guidance, the starting dose was determined to be 0.05 mg/kg, which was equivalent to one-tenth of the MTD in the most sensitive species (3.3 mg/kg in rats that corresponded to a human equivalent dose of 0.2 mg/kg after adjusting for body surface area). However, much higher starting doses could also be derived using acceptable alternative methodologies, including one-sixth of the highest non-severely toxic dose in nonrodent species (weight adjusted human equivalent, 0.37 mg/kg) or the human equivalent of an animal weight-adjusted NOAEL in the most sensitive species (0.22 mg/kg). Because of the high probability that the starting dose grossly underestimated therapeutically relevant doses, the study utilized hyper-accelerated dose titration in the first patient cohort, in which there was intra-patient dose escalation twice in the first treatment cycle, followed by accelerated dose titration in patient cohorts 2 and 3, in which there was intra-patient dose escalation between cycles 1 and 2 (Table 1). The dose-limiting toxicity (DLT) evaluation period was two cycles (8 weeks). An assessment of drug exposure between the 0.6 and 1.2 mg/kg dose levels was planned to confirm the appropriateness of dose escalation based on weight.

**Table 1:** Dose escalation scheme and relevant events (*n* = 14)^a^

Cohort						
No.	No. Pts Treated	Dose Level	Dose (mg/kg)	No. Treated Patients	No of Cycles	No. Patients with DLT
1^a^ (Hyper-accelerated Titration)	4	1	0.05	4	4	0
		2	0.15	4	4	0
		3	0.30	4	8	0
2^b^ (Accelerated Titration)	8	3	0.30	8	8	0
		4	0.60	3	8	0
3^c^ (Accelerated Titration)	2	5 6	1.2 2.0	2 -	2 -	2 -

^a^Fifteen patients were enrolled and 14 patients were treated. One patient was withdrawn per protocol due to significant neutropenia prior to VLX1570 treatment

^b^Hyper-accelerated titration cohorts with intra-patient dose escalation

^c^Accelerated titration with intra-patient dose escalation

Blood sampling for pharmacokinetic (PK) studies was performed pretreatment, immediately after the end of treatment and at 0.25, 0.5, 1, 1.5. and 3 h post-treatment on days 1 and 15. Sampling was performed pretreatment, immediately after the end of treatment on day 2. VLX1570 was assayed in plasma using a validated liquid chromatography tandem mass spectrometry method with a lower limit of quantification of 1.0 ng/mL The plasma concentration data were analyzed using the program PK-Solutions 2.0TM (Summit Research Services and Software, Montrose CO). Non-compartmental pharmacokinetic analysis was applied.

The study and all amendments were approved by the institutional review boards at participating sites, and patients gave written informed consent.

## Results

Fifteen patients were enrolled into the study, of whom 14 patients, whose relevant disease characteristics are shown in Table 2, received drug treatment. Four and eight patients, were treated with VLX1570 in cohort 1 (dose levels, 0.05, 0.10 and 0.15 mg/kg) and cohort 2 (dose levels, 0.30 and 0.60 mg/kg), respectively, with three patients in each cohort being evaluable for toxicity and the remaining patients withdrawn from study due to progressive disease. In cohort 2, three of four patients received additional VLX1570 treatment at the 0.60 mg/kg dose level. One cohort 2 patient who received four VLX1570 cycles (one cycle at 0.30 mg/kg and three cycles at 0.60 mg/kg) after developing rapid progression of an IgA kappa MM on standard therapy prior to study entry experienced improved overall functionality and constitutional symptoms, as well as modest decrements of several MM parameters compared to pretreatment baseline, including serum M protein (0.80 to 0.60 mg/dL), 24-h urine M protein excretion (184 to 81 mg); urine kappa free light chain (FLC) (114 to 91 mg/L), and kappa/lambda FLC ratio (51 to 18), consistent with stable disease. Adverse events (AEs), possibly related to VLX1570, but likely confounded by disease, included grade 1–2 fatigue, rash, nausea, and anemia; DLT was not observed.

**Table 2:** Patient Characteristics (*n* = 14)

	*n* (%)
Median age (range), y	65 (46–78)
Female	7 (50%)
Median prior lines of therapy (range)	5 (2–14)
Prior PI	14 (100%)
At least two prior PIs	13 (93%)
Prior IMiD	14 (100%)
Prior pomalidomide	12 (86%)
Double refractory (PI and IMiD)	14 (100%)
Prior daratumumab	6 (43%)
Prior high-dose melphalan with ASCT	11 (79%)

Fifteen patients were enrolled and 14 patients were treated. One patient was withdrawn due to neutropenia prior to VLX1570 treatment, as detailed previously

*ASCT* autologous stem cell transplantation, *IMiD* immunomodulatory imide drug, *PI* proteasome inhibitor

In contrast to the relative lack of AEs in cohorts 1 and 2, fatal pulmonary toxicity occurred in the first two cohort 3 patients within days of receiving two doses of VLX1570 1.2 mg/kg. The first patient, a 58-year-old female who had received extensive prior anti-MM therapy, including four proteasome inhibitors and an autologous stem cell transplantation (ASCT), had experienced a severe pulmonary event, characterized by bilateral pulmonary infiltrates that were attributed to an atypical pneumonia 10 months prior to study. On VLX1570 treatment day 3, she developed cough, fever, dyspnea, and hypoxia, and diffuse bilateral ground glass opacities on computerized tomography (CT), all of which worsened over the next week. She also developed severe anemia requiring transfusions and thrombocytopenia (platelet count nadir, 17,000/mm^3^ on day 10) without clinical or laboratory evidence of bleeding, hemolysis, or platelet consumption. Despite receiving broad spectrum antibiotics and antifungals and high-dose corticosteroids, the patient developed multi-organ failure and expired on treatment day 10. A distinct infectious etiology was not identified, but the event was initially attributed to an atypical infection, largely in consideration of her previous pulmonary event. The second patient, a 75-year-old man who had also previously received extensive anti-MM therapy, including bortezomib and carfilzomib, daratumumab, and an ASCT, developed mild transient dyspnea during his second VLX1570 treatment; a chest radiograph was unremarkable. On day 3, however, he developed progressively worsening dyspnea, hypoxia, and cough. Bilateral lung infiltrates were noted on CT and both mechanical ventilation and broad-spectrum antibiotics were begun. On day 5, high-dose corticosteroids were administered post- bronchoscopy, which did not reveal any specific findings nor positive cultures; a lung biopsy was not performed. Like the previous patient, transfusion-dependent anemia and thrombocytopenia (platelet nadir, 9000/mm3 on day 10) without clinical or laboratory evidence of bleeding, hemolysis, microangiopathy or platelet consumption, were noted. The patient’s free serum kappa light chain M-protein decreased from a pretreatment value of 52.0 to 11.8 mg/dL on day 9 despite worsening renal dysfunction, and most unfortunately, he expired on treatment day 10 with multi-organ failure. After these severe pulmonary AEs culminating in death, the study was terminated.

PK studies revealed that VLX1570 was not quantifiable at doses less than 0.3 mg/kg nor at durations longer than 3 h post-treatment at higher doses. Peak plasma concentrations (C_max_) were measured in 11 patients at the end of 31 infusions in the dosing range of 0.3–1.2 mg/kg; C_max_ values ranged from 2.0 to 68.5 ng/mL. C_max_ values did not relate to body weight. Assuming a plasma volume of 3000 mL, the magnitude of the C_max_ values indicate that the amount present in the plasma compartment at end of infusion was approximately 0.03–0.9% of the administered dose. These estimates are similar to those resulting from studies in rats and monkeys and are at least partly explained by the rapid metabolism of VLX1570 following infusion into the blood. Similarly, plasma clearance rates ranged from 8 to 62, 11 to 32, and 42 to 94 L/h/kg, respectively, at the 0.3, 0.6, and 1.2 mg/kg dose. These values are much larger than estimates for liver blood flow and glomerular filtration rate, 1.5 and 0.12 L/h/kg, respectively, again indicating that VLX1570 is rapidly metabolized following infusion. The elimination half-life ranged between 0.17 and 1.01 h.

Interindividual variability was high and clearance was rapid, with half-life values averaging 0.49 ± 0.24 h. The rapid clearance rate was much higher than the glomerular filtration rate and liver blood flow, supporting preclinical results indicating that VLX1570 is rapidly metabolized. The high intraindividual variability, level of assay quantification, and rapid clearance precluded accurate assessments of dose proportionality and weight-based dosing. Although VLX1570 concentrations and exposure in both patients with severe pulmonary toxicity were among the highest, several other patents treated at lower doses without lung toxicity had higher values.

## Discussion

Pulmonary toxicity, albeit rare, has also been reported with PIs targeting the 20S proteasome subunit [14–16]. In a recent literature review of 35 reports of pulmonary toxicity attributed to bortezomib, most events were noted to occur after the first several treatments and the mean duration from the last dose to the onset of pulmonary symptoms was 3 days; pulmonary toxicity was fatal in 37% of patients in the series [14]. Mortality did not relate to gender, prior lung disease, history of smoking, concurrent or prior treatment with corticosteroids, nor management with high-dose corticosteroids. Nonetheless, rapid clinical improvement has been reported following treatment with high-dose corticosteroids [15]. Although the underlying mechanism for PI-induced pulmonary toxicity is unclear, the withdrawal of the agents, possibly triggering rebound activation of (NF)-κB and lung inflammation, has been proposed [14]. The preferential accumulation of active drug metabolites in the lung, thereby resulting in direct damage to tissues, is an alternative hypothesis [14]. The severe lung toxicity of VXL1570 at doses at which potential antitumor effects were just beginning to be noted understates the robust, albeit nonselective, inhibitory effects of targeting the upstream 19S regulatory proteasome subunit with VLX1570. The contribution of the components in VLX1570’s formulation to the pulmonary events must also be considered, however, the excipients are not known to confer such effects.

To better understand these clinical observations, a rat model of VLX1570-induced lung toxicity has recently been developed. Rats treated with VLX1570 and a related analog developed dose-related respiratory dysfunction, as well as multifocal hyperplasia of the alveolar and, to a lesser degree, of the bronchiolar epithelium, associated with an intra-alveolar accumulation of macrophages. The effects appear to be enhanced by the PCT formulation. Further, bortezomib formulation in PCT was also associated with similar findings; thus, this model is being used to elucidate the mechanism of VLX1570-induced lung toxicity and identify DUB inhibitors with greater selective for cancer than lung tissue. Despite the severe pulmonary toxicity noted with VLX1570 in the present study, efforts directed at identifying DUB inhibitors with greater therapeutic indices appear warranted based on the unique mechanism of action, robustness of preclinical antitumor activity, and activity of the DUB inhibitors in MM resistant to PIs targeting the 20S proteasome subunit.
